# On the After-Pains of Parturition

**Published:** 1824-10

**Authors:** 


### Art. V.—*On the After-Pains of Parturition.* By Dr. Kinglake.

The after-pains of parturition are chiefly induced by uterine contraction, designed to close and secure from further bleeding the vascular openings produced after the expulsion of the fœtus, by the detachment and exclusion of the placenta. Were these pains or contractions not to occur, destructive hemorrhage would most probably ensue; it is therefore of the utmost importance that this, as well as all other ord[illegible]nances of nature in the animal economy, should obtain in the most uninterrupted and effectual manner. When these pains are efficient for the purpose destined to be answered by them, the maternal vessels, through which the fœtus derived its support during the period of utero-gestation, will be securely restrained, by the collapsed or contracted state of the uterus bringing into close contact every part of its internal surface, and thereby obliterating its cavity.

An ample provision is made in the contractility of the uterus for this salutary event, without which the birth of a child would be almost invariably the death of the mother. If this inherent contractile power be not duly exerted, the deficiency should be suitably supplied, to obviate the fatal consequence which the want of it may occasion.

The contractile power of the uterus is so strongly exerted during the period of parturient excitement, as greatly to diminish its energy. It is, in fact, incapacitated for a time for making any further effort. This is the quiescent or recruiting period, that succeeds the event of parturition. After the lapse of about half an hour, the enfeebled state of vital power acquires renewed force, and enables the uterus to resume such further contractile effort as the exclusion of the placenta, or after-birth, may require.

During the suspended exertion from dependent power, there is but little danger of any considerable effusion from unclosed vessels. In general, the placenta retains its attachment, and will not be separated until the renovated powers of the uterus shall become susceptible of being excited by its presence. At that time, the occurrence of the pains denominated after-pains is nature's effective agent for the removal of whatever may be distending the uterus, and thereby prevent such an approximation of its sides as would destroy any cavity, or unoccupied space, into which any sanguineous effusion could be deposited. The process of placental delivery by spontaneous effort, is not less natural than that of fœtal parturition, and is, of course, under equally precise regulations: they are, indeed, different stages of the same function, and one is incomplete without the other.

It would not be less premature, or preposterously officious, to attempt to remove the placenta before the contractile powers of the uterus be restored, after the temporary exhaustion that ensues the expulsion of the fœtus, than it would be, at the full period of pregnancy, forcibly to extract the child without any parturient pain. In both instances, the interference would be pernicious, and would be likely to pervert, and render ineffectual, the natural effort that may be duly made for delivery.

The pain that occurs after child-birth, and previously to the removal of the placenta, denotes the exertion of a contractile power in the uterus, which is destined to finish the process of parturition: it should, therefore, be not only tolerated, but hailed as a remedial benefit, which will at once exonerate the uterus from its remaining incumbrance, and enable it to secure against hemorrhage, by the close contact of surfaces, which, when separated, formed the uterine cavity.

The prevailing practice of employing opium to allay after-pains, is founded on an incorrect estimate of the nature and effect of those pains; and is in general inadmissible, from a probability of being injurious. It would be better that the uterus should be left to the natural influence of its contractile power to obliterate its cavity, than to resort to opiate or narcotic agency for quieting uneasy sensations, that are more likely to prove salutary than hurtful.

An uncontracted uterus, after the removal of the placenta, is a state fraught with imminent risk to the safety of the patient. The flooding that may ensue may be uncontrollable, and may, by its extent, be rapidly destructive of life. When any considerable effusion of blood continues after the exclusion of the placenta, prompt and scrupulous examination should be directed to the interior state of the uterus, knowing, if that organ had sufficiently contracted, there would be scarcely any bleeding. Coagulated masses of blood, large enough to distend the uterus, and to keep its bleeding vessels open, may be accumulated in its cavity. The hand, on these occasions, should be unhesitatingly introduced into the uterus, and the dilating contents wholly brought away, the removal of which, with the excitement produced by the manual operation, will be likely to restrain further bleeding, by inducing the degree of contraction that would prevent the possibility of its recurring.

In all cases of parturition, a sufficient interval after the birth of the child should be allowed for the uterus to recruit its exhausted strength before the placenta should be removed. When it is detached, either simultaneously with the expulsion of the fœtus or speedily after, the process is natural, and cannot be restrained or regulated: but this is not the ordinary course; the delay is often indefinite, and occasionally too long to admit of safely leaving the event altogether to a spontaneous termination. Under these circumstances, the utility of scientific interference is evinced, as it accomplishes with certainty that which could not be decisively effected without such assistance. After an hour shall have elapsed, a sufficient interval will have been afforded for the necessary renewal of uterine energy; and, if it be not exerted, it must be owing to a deficiency of power, that may require adventitious excitement. This would be furnished by employing such measured and regulated force in removing the placenta, as would at once effect its detachment, and stimulate the uterus to the degree of contraction that would close the vessels that had been opened by the placental separation.

After this desirable object has been attained, it would be much safer to leave the uterus to the stimulant influence of the slight pain resulting from the change of circumstances, and such incidental excitement as may have occurred, than to aim at subduing it by opiate or narcotic aid. If the after-pain be so excessive as to endanger inflammation, it should be appeased; but its moderate existence should be regarded rather as salutary than morbid.

Much of the difficulty attending hemorrhage after parturition, may be obviated by strictly bandaging the abdomen immediately after delivery, which will both facilitate the contraction of the uterus, and check any occurring tendency to faintness arising from the abrupt removal of the mechanical support which had been afforded by the pregnant state to the adjacent abdominal viscera. When the pulse sinks into extreme smoothness, with an intermitting or faultering action, and the countenance and cutaneous surface assume a deathly paleness, whatever precautions may have been taken against the event, it is reasonable to presume that blood is largely escaping from the sanguiferous vessels. If it should not be apparent by any external discharge, still it may be effusing into the cavity of the uterus, which, though compressed, may not have contracted sufficiently to bring its sides into close contact with each other. Under such circumstances, the shuddering induced by the sudden application of cold water, to which may be added one-fourth part of vinegar, to the region of the abdomen and loins, and the more permanent sedative influence of continued topical cold in these situations, may serve to restrain the flooding, so as to prevent its going to a destructive extent. On these occasions, it is not enough to know and deplore the menacing difficulty, but active endeavours should be made to stem the dangerous current; and if the heart be yielding to a state of inanity and want of excitement, powerful stomachic stimulants, such as brandy, spirit of turpentine, &c. should be freely administered, with a view to counteract the dying debility and inertness to which vital power has been reduced.

Two objects should never be disregarded in the management of parturient patients: the one is, not to suffer excessive after-pain to prevail, lest it should produce uterine and peritoneal inflammation, by which puerperal disease is usually characterized; and the other is, to hasten, rather than check, the natural degree of after-pain, as being indispensably necessary to induce an effectual contraction of the uterus, by which alone destructive flooding can be securely obviated. Happily for the safety of female life in its parturient function, the provisions and resources of nature are fully equal to all the great exigencies of the occasion; but exceptions occur *(exceptio firmat regulam)* in which assistance may be required; and advantageous will it be in these instances for the patient, if the needful aid be rendered in strict accordance with existing necessities, by which alone can effectual benefit be afforded.

Public teachers, as well as practitioners, of the obstetric art, have differed in opinion relative to the removal of the placenta.

Whether its expulsion was not as much the natural office of the uterus as that of the exclusion of the fœtus, has been gravely debated; and the proposition has been affirmed and denied, according to the different views and convictions of the disputants on the subject. The placenta cannot be justly regarded as a *noli me tangere*; but its temporary station, and seasonable detachment, are objects of important concern in the practice of midwifery. Neither premature assistance should be obtruded, nor should the necessary aid be too long delayed. Both in the one case and in the other, destructive flooding may be incurred. The middle course, as on most other occasions, is the safest line of conduct: "*in medio tutissimus ibis*." When the event is not anticipated by an unusually precipitate course of uterine contraction, the interval of from half an hour to one hour after the birth of the child would be the eligible period for assisting the separation of the placenta, by gently drawing the funis. A slight degree of force in this way, is ordinarily sufficient to induce the necessary contraction for effecting its prompt expulsion; when abdominal pressure, and the moderate pain that ensues the detachment, will cause such an approximation of the sides of the uterus, as will leave no unoccupied space into which any considerable effusion could be deposited.

A rapid transition from life to death from uterine flooding, without any consciousness of ailment, is an appalling liability. The event, however, is often occurring; and it may be affirmed, without fear of contradiction, that it is generally attributable to some incautious proceeding with respect to the period of removing the placenta; and to the state of the uterus, in regard to its cavity being open and distended, or effectually closed. No patient should be left, after the delivery of the placenta, whilst flooding remains, without examining the cavity of the uterus, to ascertain if there be coagula or other substances in it, preventing a close mutual contact of its internal surfaces: in which case, the obstacle to this desirable state should be removed; which, with moderate pressure over the region of the abdomen, also topical cold in that situation, if necessary, and the adoption of a general sedative or anti-stimulant treatment, will be likely to insure a favourable termination to every occurring difficulty.

*Taunton; July 14th*, 1824.

