# Corrections

**Published:** 1824-11

**Authors:** 


*** In complying with Dr. K.'s request of inserting these corrections here, instead of at the end of the Number, we must be allowed to say, that the inaccuracies arose from the difficulty of decyphering his writing.

| | | | |
|---|---|---|---|
| Page 291, | line 7th, | *for* ordonnances, | *read* ordinances. |
| — 292, | — 15th, | — dependent, | — deficient. |
| — 294, | — 9th, | — smoothness, | — smallness. |
| — 295, | — 25th, | — recurred, | — occurred. |

